# Supplementary material for: A Neyman-Pearson Framework for Modeling Cellular Decision Making Using Single-Cell TNF–NF-κB Signaling Data
Source: bioRxiv. 2025 Dec 31:2025.12.30.696266. Preprint. [Version 1] doi: 10.64898/2025.12.30.696266 (PMC12776388; doi:10.64898/2025.12.30.696266)
Supplement: 1 [file NIHPP2025.12.30.696266v1-supplement-1.pdf]

bioRxiv preprint doi: https://doi.org/10.64898/2025.12.30.696266; this version posted December 31, 2025. The copyright holder for this preprint (which was not certified by peer review) is the author/funder, who has granted bioRxiv a license to display the preprint in perpetuity. It is made available under aCC-BY 4.0 International license.

SUPPLEMENTARY MATERIALS

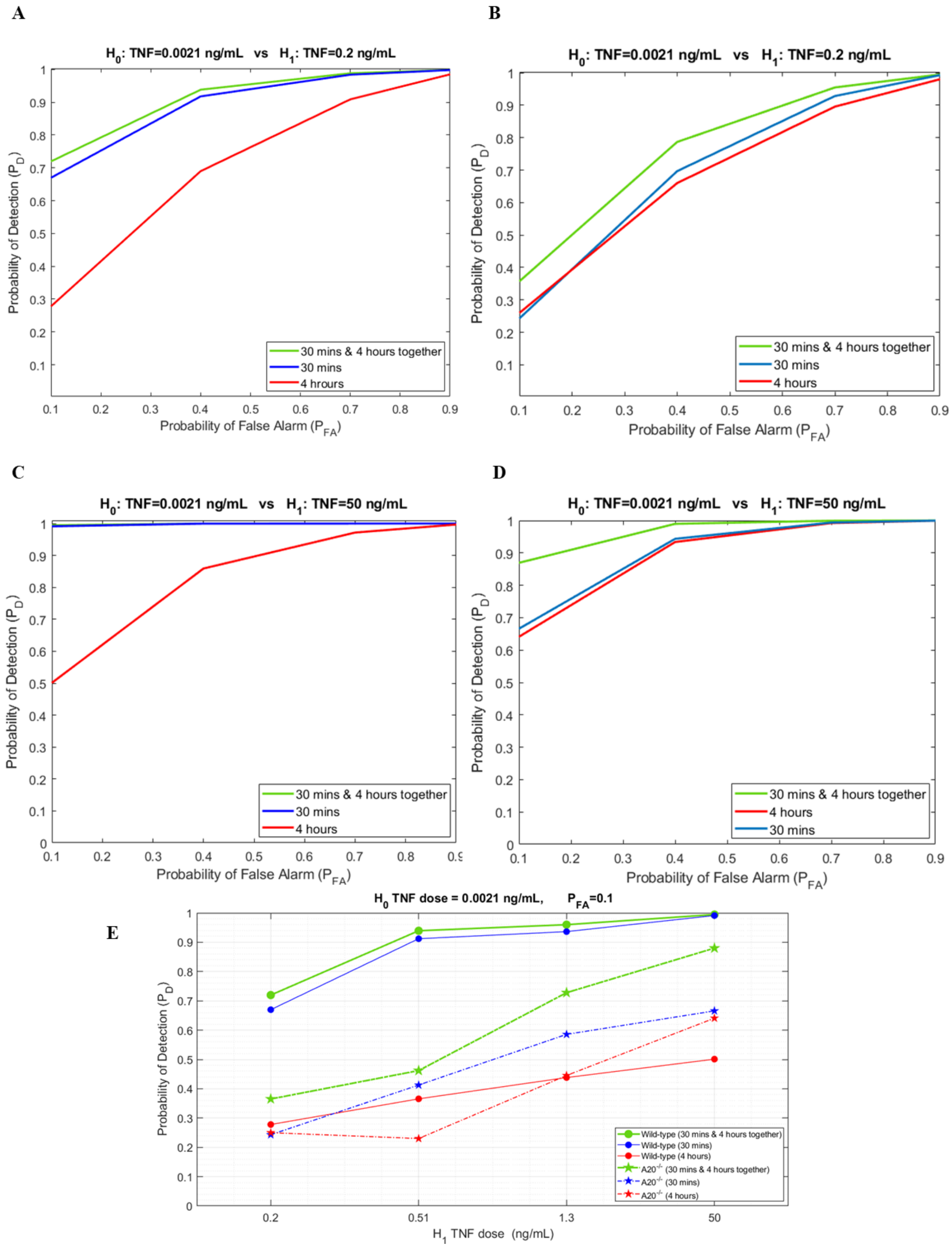

Figure S1. Receiver operating characteristic (ROC) curves for TNF–NF-κB signaling pathway evaluated using Neyman-Pearson theorem when low TNF H_0 is 0.0021 ng/mL. A) Wild-type cells result for high TNF H_1 equal to 0.2 ng/mL. B) A20$^{-/-}$ cells results for high TNF H_1 equal to 0.2 ng/mL. C) Wild-type cells result for high TNF H_1 equal to 50 ng/mL. D) A20$^{-/-}$ cells results for high TNF H_1 equal to 50 ng/mL. E) Probability of Detection vs. various TNF levels for both wild-type and A20$^{-/-}$ cells at different time points.

bioRxiv preprint doi: https://doi.org/10.64898/2025.12.30.696266; this version posted December 31, 2025. The copyright holder for this preprint (which was not certified by peer review) is the author/funder, who has granted bioRxiv a license to display the preprint in perpetuity. It is made available under aCC-BY 4.0 International license.

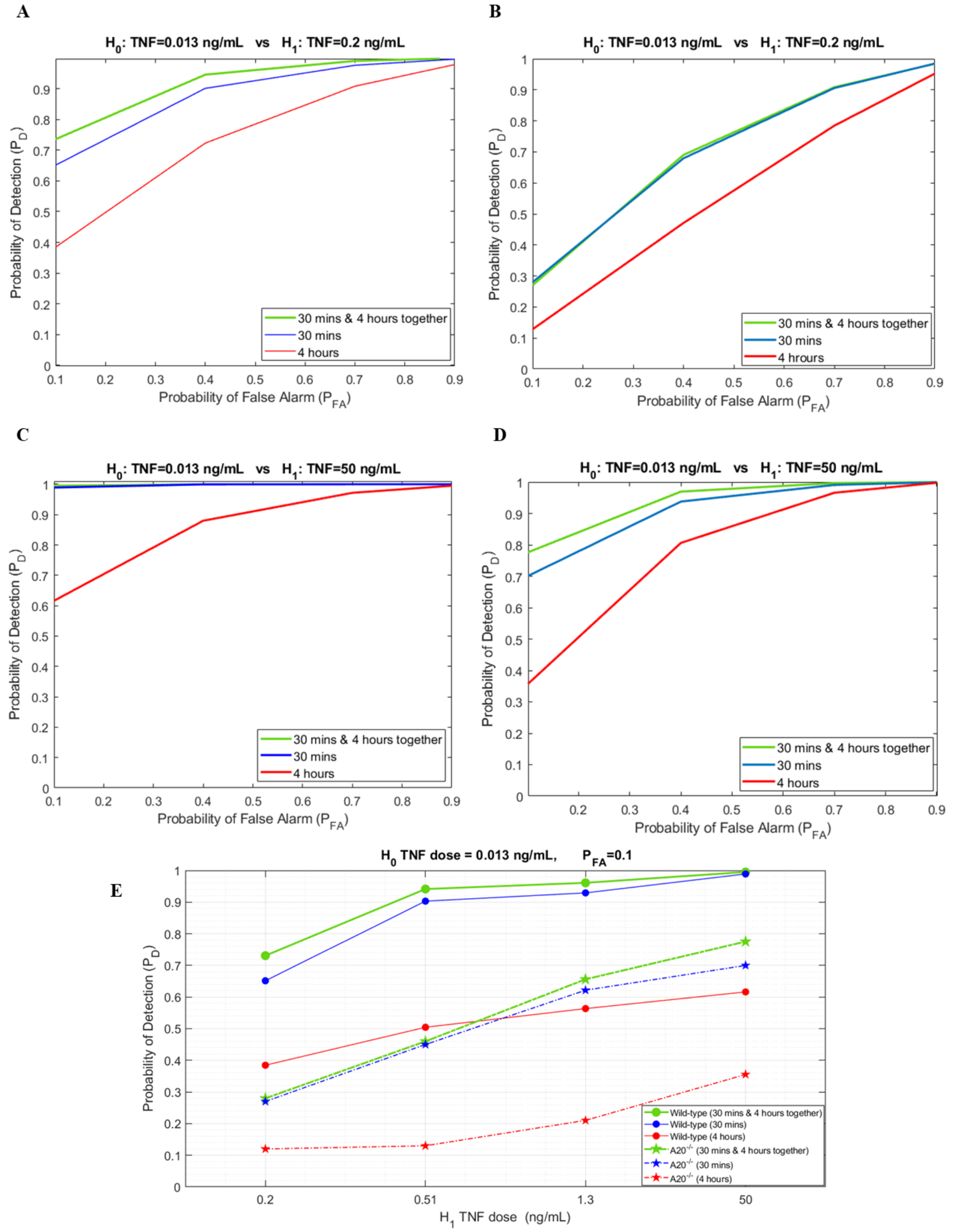

Figure S2. Receiver operating characteristic (ROC) curves for TNF–NF-κB signaling pathway evaluated using Neyman-Pearson theorem when low TNF H_0 is 0.013 ng/mL. A) Wild-type cells result for high TNF H_1 equal to 0.2 ng/mL. B) A20$^{-/-}$ cells results for high TNF H_1 equal to 0.2 ng/mL. C) Wild-type cells result for high TNF H_1 equal to 50 ng/mL. D) A20$^{-/-}$ cells results for high TNF H_1 equal to 50 ng/mL. E) Probability of Detection vs. various TNF levels for both wild-type and A20$^{-/-}$ cells at different time points.

bioRxiv preprint doi: https://doi.org/10.64898/2025.12.30.696266; this version posted December 31, 2025. The copyright holder for this preprint (which was not certified by peer review) is the author/funder, who has granted bioRxiv a license to display the preprint in perpetuity. It is made available under aCC-BY 4.0 International license.

Mean Vectors and Covariance Matrices after Eigenvalue Decomposition for Various Scenarios:

$$\mathbf{X} = \begin{bmatrix} X_1 \\ X_2 \end{bmatrix} \sim \begin{cases} \mathrm{N}\ (\mathbf{0}, \mathbf{I}) & \mathrm{H}_0 \\ \mathrm{N}\ (\mathbf{m}, \mathbf{\Lambda}) & \mathrm{H}_1 \end{cases} \qquad \mathbf{m} = \begin{bmatrix} m_1 \\ m_2 \end{bmatrix}, \qquad \mathbf{\Lambda} = \begin{bmatrix} \lambda_1 & 0 \\ 0 & \lambda_2 \end{bmatrix}$$

A) Wild-type:

$$\begin{cases} \mathrm{H}_0: & \mathrm{TNF} = 0.0021\ \mathrm{ng/mL} \\ \mathrm{H}_1: & \mathrm{TNF} = 0.2\,\mathrm{ng/mL} \end{cases} \qquad \mathbf{m} = \begin{bmatrix} 1.7311 \\ 0.7279 \end{bmatrix}, \qquad \mathbf{\Lambda} = \begin{bmatrix} 0.9943 & 0 \\ 0 & 0.9406 \end{bmatrix}$$

$$\begin{cases} \mathrm{H}_0: & \mathrm{TNF} = 0.0021\ \mathrm{ng/mL} \\ \mathrm{H}_1: & \mathrm{TNF} = 50\,\mathrm{ng/mL} \end{cases} \qquad \mathbf{m} = \begin{bmatrix} 3.5776 \\ 1.3014 \end{bmatrix}, \qquad \mathbf{\Lambda} = \begin{bmatrix} 0.9443 & 0 \\ 0 & 0.9589 \end{bmatrix}$$

$$\begin{cases} \mathrm{H}_0: & \mathrm{TNF} = 0.0052\ \mathrm{ng/mL} \\ \mathrm{H}_1: & \mathrm{TNF} = 0.2\,\mathrm{ng/mL} \end{cases} \qquad \mathbf{m} = \begin{bmatrix} 1.8846 \\ 1.0397 \end{bmatrix}, \qquad \mathbf{\Lambda} = \begin{bmatrix} 1.1453 & 0 \\ 0 & 0.9649 \end{bmatrix}$$

$$\begin{cases} \mathrm{H}_0: & \mathrm{TNF} = 0.0052\ \mathrm{ng/mL} \\ \mathrm{H}_1: & \mathrm{TNF} = 50\,\mathrm{ng/mL} \end{cases} \qquad \mathbf{m} = \begin{bmatrix} 3.9181 \\ 1.6206 \end{bmatrix}, \qquad \mathbf{\Lambda} = \begin{bmatrix} 1.1453 & 0 \\ 0 & 0.9836 \end{bmatrix}$$

$$\begin{cases} \mathrm{H}_0: & \mathrm{TNF} = 0.013\ \mathrm{ng/mL} \\ \mathrm{H}_1: & \mathrm{TNF} = 0.2\,\mathrm{ng/mL} \end{cases} \qquad \mathbf{m} = \begin{bmatrix} 1.6998 \\ 0.9474 \end{bmatrix}, \qquad \mathbf{\Lambda} = \begin{bmatrix} 1.0831 & 0 \\ 0 & 1.2677 \end{bmatrix}$$

$$\begin{cases} \mathrm{H}_0: & \mathrm{TNF} = 0.013\ \mathrm{ng/mL} \\ \mathrm{H}_1: & \mathrm{TNF} = 50\,\mathrm{ng/mL} \end{cases} \qquad \mathbf{m} = \begin{bmatrix} 3.6773 \\ 1.6132 \end{bmatrix}, \qquad \mathbf{\Lambda} = \begin{bmatrix} 1.0831 & 0 \\ 0 & 1.2923 \end{bmatrix}$$

bioRxiv preprint doi: https://doi.org/10.64898/2025.12.30.696266; this version posted December 31, 2025. The copyright holder for this preprint (which was not certified by peer review) is the author/funder, who has granted bioRxiv a license to display the preprint in perpetuity. It is made available under aCC-BY 4.0 International license.

B) A20$^{-/-}$:

$$\begin{cases} H_0: & \text{TNF} = 0.0021 \text{ ng/mL} \\ H_1: & \text{TNF} = 0.2 \text{ ng/mL} \end{cases} \qquad \mathbf{m} = \begin{bmatrix} 0.6768 \\ 0.6515 \end{bmatrix}, \qquad \boldsymbol{\Lambda} = \begin{bmatrix} 0.6650 & 0 \\ 0 & 0.9106 \end{bmatrix}$$

$$\begin{cases} H_0: & \text{TNF} = 0.0021 \text{ ng/mL} \\ H_1: & \text{TNF} = 50 \text{ ng/mL} \end{cases} \qquad \mathbf{m} = \begin{bmatrix} 1.5959 \\ 1.5839 \end{bmatrix}, \qquad \boldsymbol{\Lambda} = \begin{bmatrix} 0.7148 & 0 \\ 0 & 0.7772 \end{bmatrix}$$

$$\begin{cases} H_0: & \text{TNF} = 0.0052 \text{ ng/mL} \\ H_1: & \text{TNF} = 0.2 \text{ ng/mL} \end{cases} \qquad \mathbf{m} = \begin{bmatrix} 0.6241 \\ 0.2430 \end{bmatrix}, \qquad \boldsymbol{\Lambda} = \begin{bmatrix} 0.8085 & 0 \\ 0 & 0.8889 \end{bmatrix}$$

$$\begin{cases} H_0: & \text{TNF} = 0.0052 \text{ ng/mL} \\ H_1: & \text{TNF} = 50 \text{ ng/mL} \end{cases} \qquad \mathbf{m} = \begin{bmatrix} 1.6375 \\ 1.1642 \end{bmatrix}, \qquad \boldsymbol{\Lambda} = \begin{bmatrix} 0.8689 & 0 \\ 0 & 0.7587 \end{bmatrix}$$

$$\begin{cases} H_0: & \text{TNF} = 0.013 \text{ ng/mL} \\ H_1: & \text{TNF} = 0.2 \text{ ng/mL} \end{cases} \qquad \mathbf{m} = \begin{bmatrix} 0.6833 \\ 0.0884 \end{bmatrix}, \qquad \boldsymbol{\Lambda} = \begin{bmatrix} 0.8699 & 0 \\ 0 & 0.7467 \end{bmatrix}$$

$$\begin{cases} H_0: & \text{TNF} = 0.013 \text{ ng/mL} \\ H_1: & \text{TNF} = 50 \text{ ng/mL} \end{cases} \qquad \mathbf{m} = \begin{bmatrix} 1.7345 \\ 0.9326 \end{bmatrix}, \qquad \boldsymbol{\Lambda} = \begin{bmatrix} 0.9349 & 0 \\ 0 & 0.6373 \end{bmatrix}$$
